# Supplementary material for: The genome sequence of the common dung beetle, Aphodius fimetarius (Linnaeus, 1758) (Coleoptera: Scarabaeidae)
Source: Wellcome Open Res. 2025 Sep 18;10:519. [Version 1] doi: 10.12688/wellcomeopenres.24863.1 (PMC12547384; doi:10.12688/wellcomeopenres.24863.1)
Supplement: Peer review history

# Reviewer report: Reviewer response for version 1

**DOI:** 10.21956/wellcomeopenres.27387.r133834

**Published:** 2025-10-22

**Authors:** Arun Arumugaperumal

**Affiliations:** 1 Department of Biotechnology, Rajalakshmi Engineering College, Thandalam, Chennai, Tamil Nadu, 602105, India

The data note describes the whole genome sequencing of the dung beetle *Aphodius fimetarius*. The genome has been assembled using a combination of PacBio long-read sequencing and Hi-C sequencing. The assembly size reported was 1,343.38 Mb, spread among 11 chromosomes. Since a male specimen was used, the X and Y chromosomes have been captured. The mitogenome was also assembled, which is of size 22.01 kb. The other member of the same genus, *Aphodius granarius,* has a genome size of 397.20 Mb [1]. It would be interesting to study the difference in the genome sizes as the genome sequence is made available now. BUSCO completeness of 99.1% indicates that the genome is a near-complete one as per the sequences available in the database.

 Figure 3 can be cropped to eliminate the unwanted regions.

 In Figure 6, the link provided is not showing blobs. It is showing squares instead. If the authors want to modify the link, they can do it at this stage.

 If possible, a link to the annotation of the genome can be updated in the manuscript.

 The article can be indexed.

Are sufficient details of methods and materials provided to allow replication by others?

Yes

Is the rationale for creating the dataset(s) clearly described?

Yes

Are the datasets clearly presented in a useable and accessible format?

Yes

Are the protocols appropriate and is the work technically sound?

Yes

Reviewer Expertise:

Bioinformatics; Genomics

I confirm that I have read this submission and believe that I have an appropriate level of expertise to confirm that it is of an acceptable scientific standard.

## References

[1] : The genome sequence of a dung beetle, Aphodius (Calamosternus) granarius Linnaeus, 1767. *Wellcome Open Research* .2025;10: 10.12688/wellcomeopenres.24306.2 10.12688/wellcomeopenres.24306.2 41103341 PMC12521904

# Reviewer report: Reviewer response for version 1

**DOI:** 10.21956/wellcomeopenres.27387.r133842

**Published:** 2025-09-27

**Authors:** Feng Zhang

**Affiliations:** 1 Nanjing Agricultural University, Nanjing, China

This manuscript presents a high-quality, chromosome-level genome assembly for *Aphodius fimetarius*, representing the first whole-genome sequence for this ecologically important dung beetle species. The technical approach is sound and follows established best practices. However, several aspects require improvement to enhance the scientific rigor and interpretative value of this work.

  

1. **Inadequate Discussion of Genome Quality Limitations**

 

1. The k-mer completeness of only 68.45% for the primary assembly falls significantly below the 95% benchmark standard, yet this critical limitation receives insufficient attention in the manuscript. The authors should provide a thorough analysis of the causes underlying the low k-mer completeness in the Results section and discuss the potential relationship between this metric and the high repeat content (59.31%)

 

1. **Methodological Details Require Clarification**

 

1. The moderate heterozygosity level (2.13%) identified by GenomeScope2 analysis warrants discussion of its impact on assembly strategy
2. The manual curation process involving 37 breaks and 53 joins needs clearer justification and criteria
3. More detailed explanation of scaffold validation procedures using Hi-C data

 

1. **Writing and Presentation Issues**

 

1. The Abstract should explicitly state that this is the first chromosome-level genome for the species
2. The extensive discussion of *A. pedellus* in the Background section is overly detailed; focus on genomically relevant aspects
3. Add a concluding section discussing future applications in ecological and evolutionary research

This manuscript presents a high-quality, chromosome-level genome assembly for *Aphodius fimetarius*, representing the first whole-genome sequence for this ecologically important dung beetle species. The technical approach is sound and follows established best practices. However, several aspects require improvement to enhance the scientific rigor and interpretative value of this work.

  

1. **Inadequate Discussion of Genome Quality Limitations**

 

1. The k-mer completeness of only 68.45% for the primary assembly falls significantly below the 95% benchmark standard, yet this critical limitation receives insufficient attention in the manuscript. The authors should provide a thorough analysis of the causes underlying the low k-mer completeness in the Results section and discuss the potential relationship between this metric and the high repeat content (59.31%)

 

1. **Methodological Details Require Clarification**

 

1. The moderate heterozygosity level (2.13%) identified by GenomeScope2 analysis warrants discussion of its impact on assembly strategy
2. The manual curation process involving 37 breaks and 53 joins needs clearer justification and criteria
3. More detailed explanation of scaffold validation procedures using Hi-C data

 

1. **Writing and Presentation Issues**

 

1. The Abstract should explicitly state that this is the first chromosome-level genome for the species
2. The extensive discussion of *A. pedellus* in the Background section is overly detailed; focus on genomically relevant aspects
3. Add a concluding section discussing future applications in ecological and evolutionary research

Are sufficient details of methods and materials provided to allow replication by others?

Yes

Is the rationale for creating the dataset(s) clearly described?

Yes

Are the datasets clearly presented in a useable and accessible format?

Yes

Are the protocols appropriate and is the work technically sound?

Yes

Reviewer Expertise:

genome

I confirm that I have read this submission and believe that I have an appropriate level of expertise to confirm that it is of an acceptable scientific standard.
